# Quantitative Determination of Thiabendazole in Soil Extracts by Surface-Enhanced Raman Spectroscopy

**DOI:** 10.3390/molecules23081949

**Published:** 2018-08-05

**Authors:** Pengcheng Nie, Tao Dong, Shupei Xiao, Lei Lin, Yong He, Fangfang Qu

**Affiliations:** 1 College of Biosystems Engineering and Food Science, Zhejiang University, Hangzhou 310058, China; npc2012@zju.edu.cn (P.N.); 21613052@zju.edu.cn (T.D.); 180312@zju.edu.cn (S.X.); linlie@zju.edu.cn (L.L.); ffqu@zju.edu.cn (F.Q.); 2 Key Laboratory of Spectroscopy Sensing, Ministry of Agriculture, China; 3 State Key Laboratory of Modern Optical Instrumentation, Zhejiang University, Hangzhou 310058, China

**Keywords:** TBZ, soil, density functional theory, surface-enhanced Raman spectroscopy, gold nanoparticle, PLS, biPLS

## Abstract

Thiabendazole (TBZ) is widely used in sclerotium blight, downy mildew as well as root rot disease prevention and treatment in plant. The indiscriminate use of TBZ causes the excess pesticide residues in soil, which leads to soil hardening and environmental pollution. Therefore, it is important to accurately monitor whether the TBZ residue in soil exceeds the standard. For this study, density functional theory (DFT) was used to theoretically analyze the molecular structure of TBZ, gold nanoparticles (AuNPs) were used to enhance the detection signal of surface-enhanced Raman spectroscopy (SERS) and the TBZ residue in red soil extracts was quantitatively determined by SERS. As a result, the theoretical Raman peaks of TBZ calculated by DFT were basically consistent with the measured results. Moreover, 784, 1008, 1270, 1328, 1406 and 1576 cm^−1^ could be determined as the TBZ characteristic peaks in soil and the limits of detection (LOD) could reach 0.1 mg/L. Also, there was a good linear correlation between the intensity of Raman peaks and TBZ concentration in soil (784 cm^−1^: *y* = 672.26*x* + 5748.4, *R*^2^ = 0.9948; 1008 cm^−1^: *y* = 1155.4*x* + 8740.2, *R*^2^ = 0.9938) and the limit of quantification (LOQ) of these two linear models can reach 1 mg/L. The relative standard deviation (*RSD*) ranged from 1.36% to 8.02% and the recovery was ranging from 95.90% to 116.65%. In addition, the 300–1700 cm^−1^ SERS of TBZ were analyzed by the partial least squares (PLS) and backward interval partial least squares (biPLS). Also, the prediction accuracy of TBZ in soil (*R_p_*^2^ = 0.9769, *RMSE_P_* = 0.556 mg/L, *RPD* = 5.97) was the highest when the original spectra were pretreated by standard normal variation (SNV) and then modeled by PLS. In summary, the TBZ in red soil extracts could be quantitatively determined by SERS based on AuNPs, which was beneficial to provide a new, rapid and accurate scheme for the detection of pesticide residues in soil.

## 1. Introduction

Thiabendazole (TBZ), a benzimidazole derivative which belongs to the absorption of a broad-spectrum fungicide, has been widely used in rape sclerotium blight, downy mildew and root rot disease prevention [1]. When TBZ is applied to prevention and pest control, about 60% of pesticides could be sprayed on the soil, which is harmful to the soil environment and plant growth [2]. The TBZ residue in soil could reach the level of mg/kg. Generally, traditional methods for determining pesticides in soil include high-performance liquid chromatography (HPLC) [3], gas chromatography–mass spectrometry (GC–MS) [4], ion exchange chromatography [5] and capillary electrophoresis [6], etc. Ali et al. [7] applied HPLC to the detection of the triazine pesticides (ametryn, atrazine, cyanazine and simazine) in soil. The relative standard deviation (*RSD*) and limit of detection (LOD) were in the range of 0.01–0.02 mg/L and 0.5–1.0 mg/L, respectively. Li et al. [8] achieved the determination of pesticides in soil by liquid-phase microextraction and gas chromatography-mass spectrometry. The results showed that LOD were between 0.05 and 0.1 μg/g with GC-MS analysis under selected-ion monitoring (SIM). Giovanni et al. [9] achieved the detection and quantitation of sulfonylurea herbicides in soil at the ppb level by capillary electrophoresis. The results suggested that the recovery of each herbicide was higher than 80% and the LOD was 10 ppb. Although the sensitivities of these methods are high, the developments of these methods were limited by the cumbersome pre-test, time-consuming detection, inconvenient instrument, expensive reagents and other shortcomings [10].

Surface-enhanced Raman spectroscopy (SERS) is a technique that enhances the intensity of Raman signals with increasing orders of magnitude. Its compound molecule signal can be enhanced in geometric multiple when it was adsorbed on some nanoscale rough metals surface (such as gold, silver and copper) [11]. Besides, SERS has the advantages of simple pretreatment, convenient equipment and fast detection speed, which is suitable for rapid screening of molecule substances [12]. In the field of agricultural TBZ residue detection, several scholars have carried out relevant researches. As for the analysis of TBZ Raman peaks, Lin et al. [13] combined SERS with silver nanoparticle technique to analyze the Raman peaks of TBZ. It was indicated that the characteristic Raman peaks at 782, 1012, 1284, 1450 and 1592 cm^−1^ belonged to the TBZ pesticides. Luo et al. [14] achieved the rapid detection of phosmet and TBZ residues in apples using SERS coupled with gold nanoparticles. The results suggested that the minimum detectable concentration was 0.5 mg/g for phosmet and 0.1 mg/g for TBZ. In addition, rapid detection of TBZ has showed a great significance in agriculture production. Feng et al. [15] combined molecularly imprinted polymers with SERS to determine trace amount of TBZ in orange juice. The whole detection took only 23 min with 4 ppm LOD for TBZ. He et al. [16] quantitatively detected the TBZ residues of apple using a surface swab capture method followed by SERS, which indicated that the swab-SERS method was simple, sensitive and rapid (10 min). Beyond these, the miniaturization and portability of Raman detection has attracted more and more attention. Although the above researches achieved the purpose of pesticide residue detection in agricultural products by SERS, the effect of matrix substances could not be fully removed and the detection limit of these methods should be further improved as well as the accuracy and stability. Moreover, there are few reports on the quantitative detection of TBZ residues in soil with the combination of SERS and chemometric method.

At present, there is no report on the use of SERS technology to detect TBZ pesticides in red soil extracts. Hence, the main purpose of this study is to apply density functional theory (DFT) to calculate TBZ molecule, optimize its structure and find its characteristic peaks. Beside this, we intended to find suitable and modified gold nanoparticles (AuNPs) to enhance the SERS detection signal as well as quantitatively determine TBZ residue in red soil extracts combined with chemometric methods, which was capable of providing a new, rapid, accurate and reliable scheme for the detection of pesticides residues in soil.

## 2. Results and Discussion

### 2.1. The TBZ Molecule and Its Assignment of Raman Peaks

**Figure 1:**
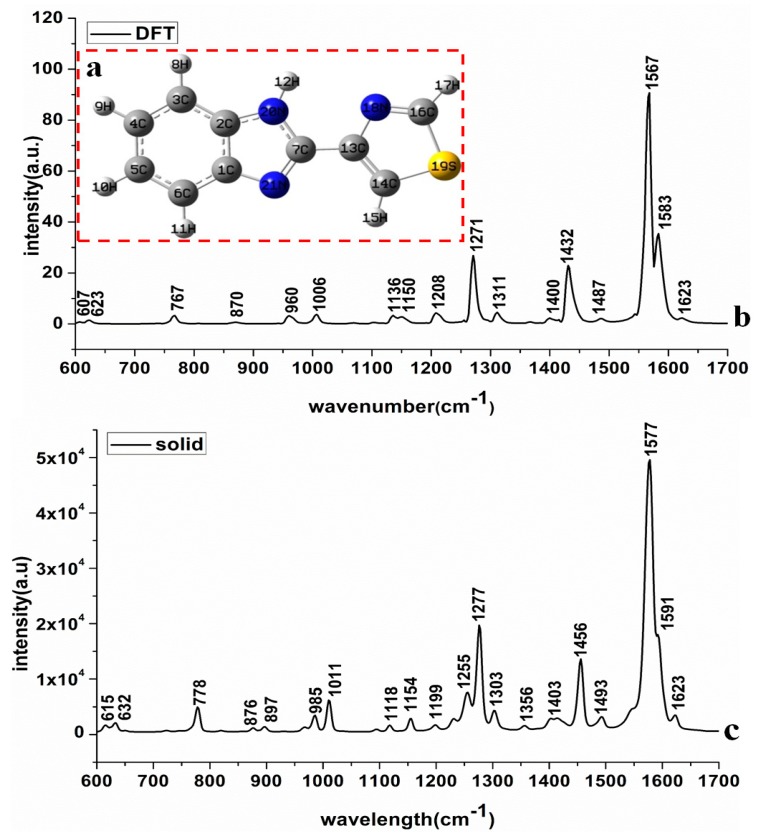
(**a**) Simulated molecular structure of Thiabendazole (TBZ) by density functional theory (DFT); (**b**) the theory calculation by DFT; (**c**) Raman spectroscopy (RS) of TBZ solid.

TBZ (molecular formula: C10H7N3S), containing benzimidazole and thiazole rings, is mainly composed of C-N, C=N, C=C, C-C, C-H, C-S and N-H groups. DFT can describe the ground-state physical properties of atoms and molecules, which is a common method for molecular geometry optimization and frequency vibration calculation [17]. In this paper, DFT was applied to calculate TBZ molecule and optimize its structure in Gaussian.v09 software. In this software, the vibrational form of relevant chemical bonds calculated by Hartree-Fock wave function can be obtained [18]. Figure 1a shows the molecular structure of TBZ, Figure 1b is the TBZ Raman spectroscopy (RS) simulated by DFT and Figure 1c is the RS of solid TBZ.

As seen in Figure 1, DFT-calculated Raman peaks were basically consistent with the experiment-detected Raman peaks of TBZ. Although some Raman peaks (960, 1135, 1432 cm^−1^) calculated by DFT differed from those in RS, their peak intensity was weak and the Raman shifts (less than 20 cm^−1^) were within a reasonable range, which indicated that DFT-calculated Raman peaks were feasible and reliable. Combining with related literature [14,19,20,21], the assignments of Raman peaks of TBZ are listed in Table 1.

(1) C-H and ring vibration: the vibration of C-H groups’ in-plane and out-of-plane bending deformation concentrated on 700–1400 cm^−1^ [21]. Figure 1b revealed that the spectral peak intensity at 778, 1011, 1255 and 1277 cm^−1^ were relatively higher than others, resulting of the strong C-H vibration among aliphatic compounds as well as the widest band range of C-H vibration [19]. Additionally, 1199 cm^−1^ and 1255 cm^−1^ belonged to the ring vibration, 1277 cm^−1^ was the synergistic effects of ring vibration and C-H groups surface vibration. Moreover, 778 cm^−1^ was the C-H outer surface bending vibration, 1011, 1118, 1303 and 1154 cm^−1^ belonged to the surface bending vibration of the C-H group [20].
(2) C=N vibration: the vibration of the C=N group is generally in the range of 1300–1700 cm^−1^, among which the spectral peak intensity at 1456, 1577 and 1591 cm^−1^ were strong. Refer to Sundaraganesan’s study [19], the C=N stretching vibration band of the ammonium imine group ranges from 1610 to 1690 cm^−1^, and the Raman peak of C=C is normally weaker than that of the C=N group. Thus, it could be confirmed that 1577, 1591 and 1623 cm^−1^ belong to the C=N stretching vibration, which was also basically consistent with the vibration peaks of C=N in TBZ identified by Cirak [22].
(3) C-C and C=C vibration: the spectral bands of C-C and C=C are usually in the range of 300–1700 cm^−1^ and these spectral peaks mainly belong to the characteristic peaks of polycyclic aromatic hydrocarbons in TBZ [21]. Among them, 615 and 632 cm^−1^ were the synergistic effects of C-C-C and S-C-N inner surface bending and deformable vibration, 876 and 897 cm^−1^ were the synergistic effects C-C-C outer surface bending deformable vibration and the surface bending vibration of C-H group. Furthermore, 1403 cm^−1^ belonged to the stretching vibration of C=C band and 1493 cm^−1^ was the synergistic effects of C=C stretching vibration and N-H inner surface bending vibration. N. Sundaraganesan et al. [19] calculated the vibrational peaks of the C=C group using density functional theory, whose calculation results were consistent with this study as well.
(4) N-H vibration: the N-H band generally belongs to the range of 300–1700 cm^−1^. In this paper, the N-H in-plane deformation vibration signal appears at 1493 cm^−1^ and this signal is relatively weak. Reference [23] showed that N-H vibration of aminobenzenes occurred near 1510 cm^−1^ and this peak was also interfaced with the C=C stretching vibration, which was in accordance with our study.
(5) C-S vibration: the C-S band vibration normally ranges from 300 to 1200 cm^−1^. Compared with the spectral peak range of the sulfur-containing compounds of C-S group and S-C-N group [24], 985 cm^−1^ belonged to the C-S stretching vibration, which was consistent with the experimental results obtained by Kim et al. [20].

### 2.2. Comparison of SERS and Raman Spectrum of TBZ

**Figure 2:**
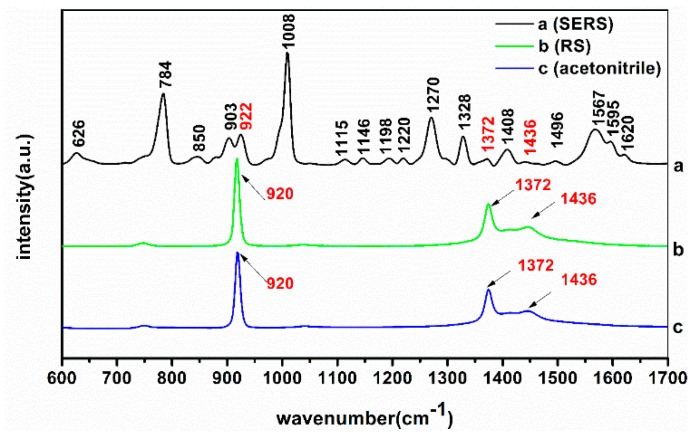
(**a**) Surface-enhanced Raman spectroscopy (SERS) of TBZ solution; (**b**) RS of TBZ solution; (**c**) RS of acetonitrile.

In order to verify the necessity of using AuNPs reinforcement, the SERS and RS of TBZ solution (dissolved in acetonitrile) were analyzed. Figure 2a is the SERS of TBZ, Figure 2b is the RS of TBZ and Figure 2c is the RS of acetonitrile. The assignments of SERS peaks of TBZ are listed in Table 1.

The RS characteristic peaks of TBZ solution were only at 920, 1372 and 1436 cm^−1^ while all these belonged to the characteristic peaks of acetonitrile. Compared with the RS of TBZ solution, there were several strong and dense Raman signal peaks in the SERS of TBZ, which indicated that the vibration of the functional groups in TBZ molecule had been greatly enhanced after mixing TBZ solution with the modified AuNPs [25]. In the SERS of TBZ, the intensity of Raman peak at 784, 903, 1008, 1270, 1328, 1408 and 1567 cm^−1^ were stronger than others. Combined with the analysis in Section 2.2, it was evident that 784 and 1328 cm^−1^ belonged to the surface bending vibration of the C-H group, 903 cm^−1^ were the synergistic effects C-C-C outer surface bending deformable vibration and the C-H band surface bending vibration, 1008 cm^−1^ belonged to the C-H band inner surface bending vibration of the C-H band, 1277 cm^−1^ was the synergistic effects of ring vibration and C-H band surface vibration and 1403 cm^−1^ belonged to the stretching vibration of C=C band.

### 2.3. Gold Nanoparticles and Its Spectral Analysis

**Figure 3:**
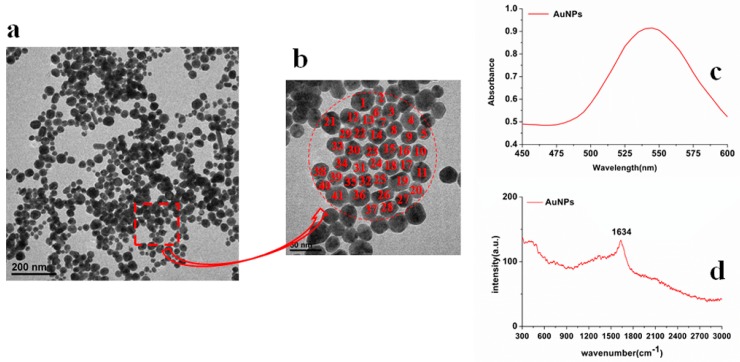
Transmission electron microscopy (TEM) images of: (**a**) AuNPs (200 nm scale) and (**b**) AuNPs (50 nm scale); (**c**) the UV/Visible spectra of AuNPs; (**d**) the SERS of AuNPs.

Gold nanoparticle is one of the unique noble metals because of the strong interaction with electromagnetic radiation and its optical properties in the visible region [26]. Therefore, the structure, particle size, UV spectrum and RS of AuNPs were analyzed. Figure 3a,b are the transmission electron microscopy (TEM) images of AuNPs in 200 nm and 50 nm scale, respectively; Figure 3c,d display the UV/Visible spectra and SERS of AuNPs orderly. Table 2 presents the average particle size of AuNPs.

As shown in Figure 3, the UV/Visible characteristic absorption peak of AuNPs was at 543 nm and there was no other absorption peak signal. In the red selected area in Figure 3b combined with Table 2, the number of AuNPs was 41, the minimum size of AuNPs was 16.7 nm, the maximum size was 36.7 nm, the average was 27.8 nm, and the standard deviation was 5.6 nm. Thus, the surface plasmon resonance of AuNPs could be stimulated more easily, resulting in the higher detection sensitivity [27]. Beside this, the Raman spectrum of AuNPs only had a faint signal at 1634 cm^−1^ (Figure 3c), suggesting that AuNPs themselves had no strong Raman characteristic peaks and did not have an interferential effect on experimental results.

### 2.4. Determination of Detection Line

**Figure 4:**
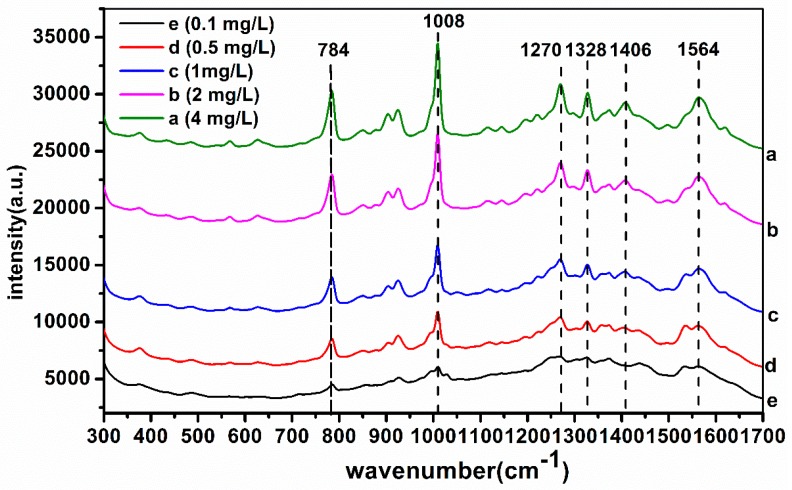
The SERS of TBZ in red soil extracts: (**a**) 4 mg/L; (**b**) 2 mg/L; (**c**) 1 mg/L; (**d**) 0.5 mg/L; (**e**) 0.1 mg/L.

In order to further determine the LOD of TBZ in red soil extracts using SERS based on AuNPs enhancement, six different TBZ concentrations in red soil extracts (4, 2, 1, 0.5, 0.1 mg/L) were collected and the corresponding SERS are shown in Figure 4.

According to Figure 4, the SERS absorbance intensity decreased gradually with the decrease of TBZ concentration at the characteristic peaks of 784, 1008, 1270, 1328, 1406 and 1564 cm^−1^. When the concentration was 0.1 mg/L, the characteristic peaks signal was weak and only the peaks of 784 and 1008 cm^−1^ could be identified. Meanwhile, when TBZ concentration was 0.5 mg/L, the six characteristic peak signals were clearly visible. Thus, it was concluded that the LOD of TBZ in red soil extracts was 0.1 mg/L for this study, using SERS based on AuNPs. Also, 784, 1008, 1270, 1328, 1406 and 1576 cm^−1^ could be determined as TBZ characteristic peaks in red soil extracts.

### 2.5. The Linear Regression Equation of TBZ in Red Soil Extracts

**Figure 5:**
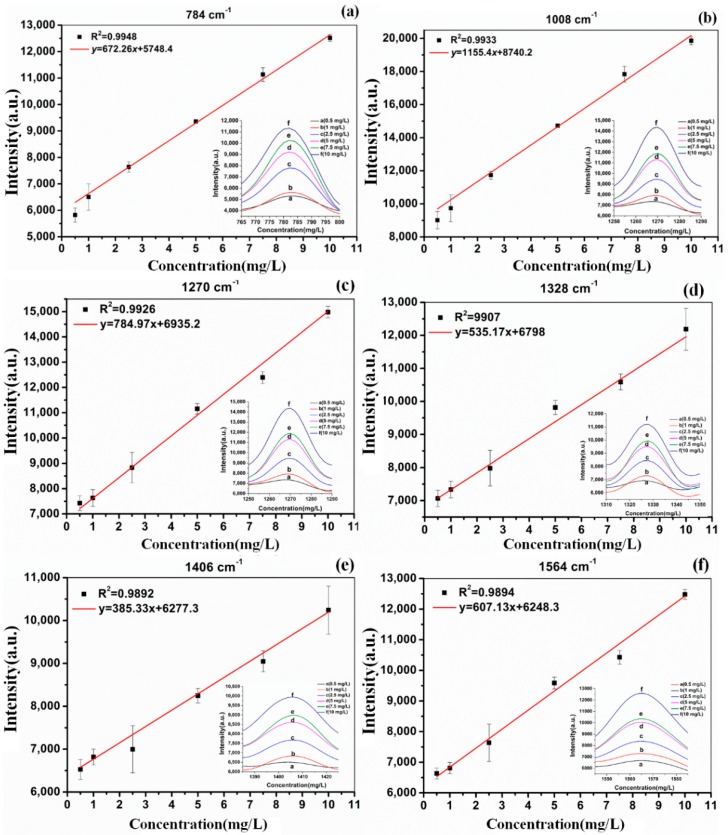
The linear regression equations between Raman peak intensity and soil TBZ concentration at different Raman peaks: (**a**) 784 cm^−1^; (**b**) 1008 cm^−1^; (**c**) 1270 cm^−1^; (**d**) 1328 cm^−1^; (**e**) 1406 cm^−1^; (**f**) 1564 cm^−1^.

Based on the above analysis, the linear regression equations between Raman peak intensity at 784, 1008, 1270, 1328, 1406 and 1576 cm^−1^ and red soil extracts with different TBZ concentrations were established (Figure 5). The TBZ concentrations of red soil extracts were 0.5, 1, 2.5, 5, 7.5 and 10 mg/L, respectively, three samples for each concentration. The abscissa represents the TBZ concentration in red soil extracts and the ordinate represents the intensity of Raman peak.

According to Figure 5, on the one hand, there was a good linear correlation between Raman peak intensity and TBZ concentration of red soil extracts solutions in each linear regression equation. At the Raman peak of 784 cm^−1^ (y = 672.26x + 5748.4; *R*^2^ = 0.9948) and 1008 cm^−1^ (y = 1155.4x + 8740.2; *R*^2^ = 0.9933), the linear correlation was superior to other Raman characteristic peaks. However, the concentration 0.5 mg/L for peaks at 784 cm^−1^ and 1008 cm^−1^ deflected from the suggested linear trend, which showed that the limit of quantification (LOQ) of these two linear models could reach 1 mg/L. On the other hand, at the Raman peak of 1270, 1328, 1406 and 1564 cm^−1^, although the linear correlation was lower than that of 784 cm^−1^ and 1008 cm^−1^, the LOQ of these four linear models could reach 0.5 mg/L. To verify the accuracy of these methods, six samples were pretreated using those six linear regression equations.

Table 3 presents the results between the true and predicted value of TBZ in red soil extracts. In the six liner model, the relative standard deviation (*RSD*) ranged from 1.36% to 10.45% and the recovery was ranging from 71.84% to 116.65%, which suggested that the predicted values were basically the same with the true values and the prediction model was feasible. Among them, the model y = 672.26x + 5748.4 (784 cm^−1^) and y = 1155.4x + 8740.2 (1008 cm^−1^) revealed the optimum prediction effect, whose *RSD* ranged from 1.36% to 8.02% and the recovery was ranging from 95.90% to 116.65%.

### 2.6. The Analysis of 300–1700 cm^−1^ SERS of Deltamethrin Pesticides in Soil

**Figure 6:**
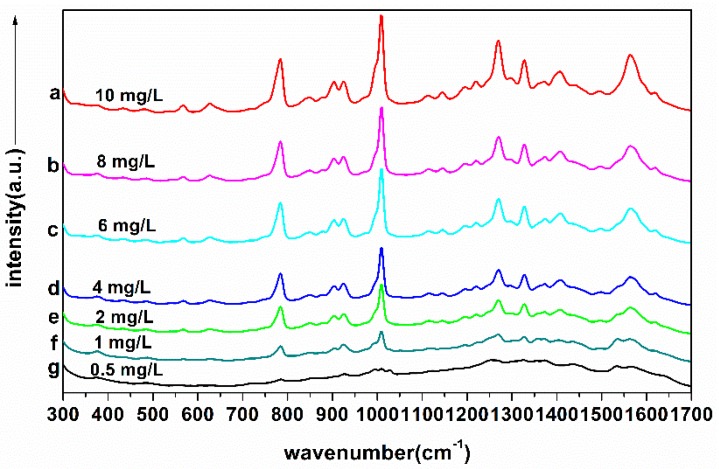
300–1700 cm^−1^ SERS spectra of seven different concentrations of TBZ in red soil extracts: (**a**) 10 mg/L; (**b**) 8 mg/L; (**c**) 6 mg/L; (**d**) 4 mg/L; (**e**) 2 mg/L; (**f**) 1 mg/L; (**g**) 0.5 mg/L.

**Figure 7:**
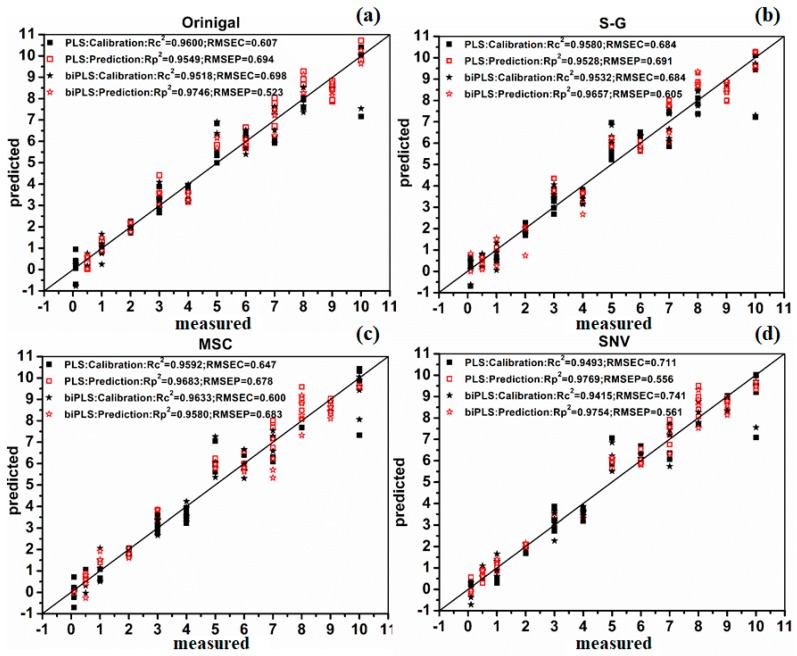
Scatter diagram of calibration set and prediction set by PLS and biPLS using different preprocessing methods: (**a**) original plot; (**b**) S-G; (**c**) MSC; (**d**) SNV.

Considering that the Raman peaks of TBZ in red soil extracts were mainly distributed in the range of 300–1700 cm^−1^, in this paper, the 300–1700 cm^−1^ SERS of 84 samples were obtained and then pretreated by Savitzky-Golay smoothing (S-G) [28], standard normal variation (SNV) [29] and multiplicative scatter correction (MSC) [30], respectively. Meanwhile, the original and preprocessed SERS were modeled by partial least squares (PLS) [31] and backward interval partial least squares (biPLS) [32]. The sample set portioning based on joint x-y distance (SPXY) method [33] was used to divide the samples into two groups, among which 54 samples were calibrated and 30 samples were validated. The SERS of the average spectra of seven different concentrations in red soil extracts are shown in Figure 6, the PLS and biPLS scatter diagrams of calibration set and prediction set with different preprocessing methods are shown in Figure 7, the prediction performance are presented in Table 4.

It can be seen that no matter whether PLS or biPLS was applied, the *R_p_*^2^ achieved more than 0.95 and the prediction accuracy of TBZ in red soil extracts was the best (*R_p_*^2^ = 0.9769, *RMSE_P_* = 0.556 mg/L, *RPD* = 5.97) when the SERS were processed with SNV and modeled by PLS (Figure 7), revealing that the TBZ in red soil extracts could be quantitatively determined by SERS based on AuNPs. According to the PLS and biPLS model performance, on the one hand, the prediction results of biPLS model were better than PLS model. The reason might be that biPLS could better extract the characteristic variables of SERS, which reduced the amount of sub-intervals of the worst or collinear variables [32]. On the other hand, no matter which spectral preprocess methods were used, the prediction effects of SNV were the optimum both in PLS and biPLS model. The reason might be that SNV could eliminate the effects of uneven sample distribution and filling density, which improved the spectral resolution, reduced standard deviation between samples and separated the main characteristic peaks for quantitative analysis [29].

### 2.7. Model Accuracy Verification

To verify the accuracy of the chemometric methods, five soil extract samples with different TBZ concentrations were pretreated using PLS model preprocessed by SNV. Table 5 presents the results between the true value and predicted value of TBZ pesticides in soil extracts, as well as *RSD* and recovery.

According to Table 5, the relative standard deviation between the true value and the predicted value ranged from 0.93 to 12% and the recovery was ranging from 89.2% to 112%. The results suggested that the predicted value was basically the same as the true value, which indicated that the rapid detection of TBZ pesticide in soil extracts by SERS was feasible.

## 3. Materials and Methods

### 3.1. Experimental Instruments and Reagents

For this study, the experimental instruments mainly included: (1) RmTracer-200-HS portable Raman spectrometer combined with a 785 nm excitation wavelength diode-stabilized stimulator (Opto Trace Technologies, Inc., Silicon Valley, CA, USA); (2) JW-1024 low-speed centrifuge (Anhui Jia Instrument and Equipment Co., Ltd., Anhui, China); (3) The FEI Tecnai G2 F20 S-TWIN transmission electron microscope (TEM, USA FEI Corporation); (4) Vortex-Genie 2/2T vortex mixer (Shanghai Ling early Environmental Protection Instrument Co., Ltd., Shanghai, China); (5) ZNCL intelligent thermostat magnetic stirrer (Zhengzhou Ya-Rong Instrument Co., Ltd., Zhengzhou, China).

Moreover, the experimental reagents included: (1) TBZ (99.8% purity, Sigma-Aldrich, Beijing, China); (2) acetonitrile (chromatographically purity, Amethyst Chemicals, Beijing, China); (3) trisodium citrate, chloroauric acid (ethylenediamine-*N*-propyls lane); (4) potassium chloride (Analytical Pure, National Standards Information Center, Beijing, China); (5) organic filter (0.22 μm, Agilent Technologies, Inc., Santa Clara, CA, USA).

### 3.2. Experimental Methods

The experimental soil samples were standard acidic red soil from the Qingyuan county, Zhejiang province, China (34°44′ N, 127°45′ E). Before the experiment, The soil was detected by high-performance liquid chromatography (HPLC). The results showed that the original soil samples were free of TBZ residues. Therefore, the concentration of the added standard solution can be considered as the final detection concentration by mixing the soil extract and the standard solution in a certain proportion.

The process of specific sample preparation was as follows. First, the red soil was collected from different points of the farmland and then mixed randomly (the sampling points were different for each mixed soil). Second, soil samples were air-dried and sieved through 80 mesh sifters (0.18 mm). Third, TBZ standard solutions with five different concentrations (1, 2, 5, 10, 20 mg/L) were prepared. Fourth, 84 soil samples were weighted and each soil sample was 10 g. Fifth, each 10 g soil sample was mixed with 5 mL ultra-pure water and put into a 50 mL centrifuge tube for 30 s vortex oscillation. Sixth, 10 mL acetonitrile (concentration: 1%) was added into the centrifuge tube and vortexed for 3 min at 400 r/min and then dealt with ultrasonic oscillation for 2 min. After resting the sample for 15 min, 3 g sodium chloride and 4 g sodium acetate were added in turn. The solution was vortexed for 1 min at 400 r/min and then put into a centrifuge for 4 min centrifugation at the speed of 10,000 r/min. Seventh, the supernatant was filtered by a 0.22 μm organic membrane. Finally, the standard TBZ solution was obtained and mixed with the soil extract in a certain proportion, thus the soil TBZ concentration of 0.1, 0.5, 1, 2, 3, 4, 5, 6, 7, 8, 9 and 10 mg/L were obtained. There were 7 samples for each concentration and 84 samples in total.

Before Raman spectra acquisition, the instrument should be calibrated using a 785 nm excitation wavelength. The parameters were set as follows: a power of 200 mw, a scanning range of 200 to 3300 cm^−1^, an optical resolution of 2 cm^−1^, an integration time of 5 s and an average spectral value of 3 times. The solid TBZ RS collection was that TBZ powder was in quartz plate with glass slides flattened and the spectra were acquired with matching microscope platform. When collecting the SERS of samples, 500 μL silver colloid, 100 μL test solution and 100 μL potassium chloride were added in turn into a 2 mL quartz bottle, then it was placed at a liquid sample pool. Potassium chloride acts as an activator, and chloride has the effect of preventing nanoparticles from agglomerating [34].

### 3.3. Gold Nano-Substrate Preparation

The trisodium citrate heating reduction method was slightly modified according to the literature for gold nanoparticle preparation [35]. The gold nanoparticle preparation process was as follows. First, a chloroauric acid solution (50 mg/L) was heated for boiling at 360 °C on a constant temperature magnetic stirrer, and then 4 mL of trisodium citrate solution (5 mg/mL) was added. Second, the mixture was stirred at 100 r/min until the gold sol changed into the color of the wine red. Third, when the solution cooled, the gold gel solution was poured into a centrifuge tube and was stored in dark at 4 °C after repeating purification. The nanoparticles prepared in this experiment were stable, and the reinforcing effect of the prepared gold colloid could maintain about 1 to 2 weeks.

### 3.4. Density Functional Theory (DFT)

The principle of DFT is the exchange-correlation functionals. The complexity of various ex-change-correlation functionals is different. It is generally divided into five levels with increasing accuracy: local density approximation (LDA), generalized gradient approximation (GGA), meta generalized gradient approximation (mGGA), hybrid, and double-hybrid functional [36]. Calculation amount of DFT is similar to that of Hartree-Fock wave function [18], but the calculation accuracy was different. Compared with traditional quantification methods, it can be applied to larger molecular systems and transition metal-containing compounds. This method not only has higher calculation accuracy and less computational complexity, but also can consider the correlation effects such as electron energy and spin to a certain degree, which is suitable for analysis of most molecular structures [37]. Among the several functions and basis sets in DFT, Perdew-Burke-Ernzerhof exchange-correlation density functional (PBE) basis set has been commonly used in the Raman spectroscopic calculation of biological molecules [38]. In this paper, PBEPBE/6-31G (d, p) was used for the theoretical simulation and calculation of TBZ molecules.

### 3.5. Spectral Preprocessing Methods

The noise caused by the equipment and the interference of the fluorescence background in the Raman signal could affect the detection results. Therefore, five pretreatment methods were applied to preprocessing the original Raman spectra in this paper [39]. For this paper, three spectral preprocessing methods were applied to dealing with the original spectra. Savitzky-Golay (S-G) smoothing [28], also known as polynomial smoothing, uses the weighted average method to quantize the data in the moving window by polynomial least squares fitting as well as emphasizing the central role of the center point. The principle of standard normal variation (SNV) [29] algorithm is that the absorbance values of each wavelength point satisfy a certain distribution in each spectra, and the spectral correction was performed according to this assumption. The basic idea of multiplicative scatter correction (MSC) [30] algorithm is to use an ideal spectra to represent all the samples, and the original spectra is corrected with the slope and intercept of the linear equation. In this paper, all data analysis was based on MATLAB R2014a (The Math-Works, Natick, MA, USA), Gaussian.v09 (Gaussian, Inc., Wallingford, CT, USA) and OMNIC v8.2 (Infrared spectrum processing software, Thermo Fisher Scientific, Waltham, MA, USA).

### 3.6. Modeling Method

Partial least squares (PLS) is a most widely used regression modeling method in spectral data analysis for its flexibility and reliability in dealing with the redundant spectral data. When PLS is applied to dealing with the spectral data, the spectral matrix is decomposed first and the main principal component variables are obtained, then the contribution rate of each principal component is calculated [31]. The flexibility of PLS makes it possible to establish a regression model in the case where the number of samples is less than the number of variables.

Backward interval partial least squares (biPLS) is a variable selection method mainly used to filter the wavelength range of PLS model and reduce the amount of sub-intervals of the worst or collinear variables [32]. The algorithm steps are mainly as follows: **a**. Divide the whole spectrum into k bands of equal width. **b**. Leave a section from the k section spectrum. Carry out the PLS regression on the remaining (k-1) section and establish the sub model of the quality to be measured. Set aside each paragraph in turn to get the k sub model. **c**. Measure the accuracy of each model by root mean square error of cross validation (RMSECV) value. Delete the reserved segment corresponding to the highest precision sub model, and take the sub model as the first base model. **d**. Leave one more section in the remaining (k-1) section of the spectrum and use the remaining (k-2) segments to model the PLS. Each section is set aside in order to obtain the (k-1) sub model to remove the reserved segments corresponding to the sub model of the minimum RMSECV value. Take the sub model as the second base model. Repeat the process until the remaining wave band. **e**. Investigate the root mean square error of prediction set (RMSEP) value of each base model according to step **b** to **d**. Select the best and minimum RMSECV among all the base models, and the corresponding interval combination is the best combination.

### 3.7. Model Evaluation Index

In this experiment, the modeling effect is evaluated by the coefficient of determination (*R*^2^), the root mean square error (*RMSE*), and the residual predictive deviation (*RPD*). The coefficient of determination *R*^2^ reflects the level of intimacy between variables, the *RMSE* reflects the accuracy of the model, and *RPD* reflects the predictive ability of the model. The higher the *RPD*, the lower the RMSE, and the closer the *R*^2^ is to 1, the better the performance of the prediction model. In this paper, R_C_^2^ and R_P_^2^ represent the coefficient of the determination of the calibration set and the prediction set respectively, while $RMSE_{c}$ and $RMSE_{p}$ represent the root mean square error of the calibration set and the prediction set respectively. In addition, the *RPD* was suggested to be at least three for agriculture applications; while 2 < *RPD* < 3 indicated a model with a good predictive ability; 1.4 < *RPD* < 2 was an intermediate model needing some improvement; and *RPD* < 1.4 indicated that the model had a poor predictive ability [40]. In addition, relative standard deviation *(RSD)* reflects the degree of discretization between individuals in the reflection group and recovery rate reflects the degree of coincidence between the results of the reaction and the true value. The recovery rate ranges closer to 100%, the lower the *RSD*, the better the reliability of the model [41].

## 4. Conclusions

We reported the determination of TBZ in red soil extracts for the first time based on SERS with AuNPs enhancement. From the preparation of red soil extract samples to the accomplishment of detection, the whole process takes only about 25 min. According to the results, we found that there was a good linear correlation between Raman peak intensity and TBZ concentration of red soil extracts, the TBZ concentration in red soil extracts was in the range of 0.1–10 mg/L and the LOD could reach 0.1 mg/L. The most favorable structures of the TBZ molecule calculated by DFT were consistent with the experiment results. The particle size of AuNPs Nano enhancer was uniform and the SERS of TBZ in red soil extracts was obviously better than the RS of TBZ in red soil extracts. Also, 300–1700 cm^−1^ SRES analysis for TBZ in red soil extracts achieved the optimum results when the original spectra were pretreated by standard normal variation (SNV) and then modeled with PLS. Overall, the SERS method with AuNPs enhancement developed through this study provides a novel, rapid and accurate approach to quantitatively determine TBZ in red soil extracts, which has the potential for broad applications in analysis of other pesticide residue in red soil extracts. Thus, we thought the further studies should be concentrated on the determination of TBZ and other pesticides in soil in agricultural fields rather than in the experimental environment.

## Figures and Tables

**Table 1:** The proposed assignment of Raman peaks of TBZ.

Calculation (cm^−1^)	Solid (cm^−1^)	SERS-Au (cm^−1^)	Assignments
607 (vs)	615 (w)	-	δ(C-C-C)opp + δ(S-C-N)ip
623 (vs)	632 (w)	626 (w)	δ(C-C-C)opp + δ(S-C-N)ip
767 (w)	778 (m)	784 (m)	δ(C-H)oop
870 (vs)	876 (w)	850 (m)	δ (C-C-C)opp + δ(C-H)opp
-	897 (w)	903 (m)	δ (C-C-C)opp + δ(C-H)opp
960 (w)	985 (m)	-	υ(C-S)
1006 (m)	1011 (m)	1008 (s)	δ(C-H)ip
1135 (w)	1118 (m)	1115 (w)	δ(C-H)ip
1150 (m)	1154 (m)	1146 (w)	δ(C-H)ip
1208 (w)	1199 (m)	1198 (w)	υ ring
-	-	1220 (w)	υ ring
-	1255 (m)	-	υ ring + δ(C-H)ip
1271 (s)	1277 (s)	1270 (m)	υ ring + δ(C-H)ip
1311 (w)	1303 (w)	1328 (m)	δ(C-H)ip
1400 (w)	1403 (w)	1408 (m)	υ(C=C)
1432 (s)	1456 (s)	-	υ(C=N)
1487 (w)	1493 (w)	1496 (w)	υ(C=C) + δ(N-H)ip
1567 (vs)	1577 (vs)	1567 (s)	υ(C=N)
1583 (s)	1591 (s)	1595 (m)	υ(C=N)
1623 (w)	1623 (w)	1620 (w)	υ(C=N)

Note: vs = very strong; s = strong; m = medium; w = weak; υ = stretching; opp = outer surface bending; ip = inner surface bending; δ = deformable vibration.

**Table 2:** The average particle size of AuNPs.

Types	Number	Min (nm)	Max (nm)	Average (nm)	Standard Deviation (nm)
AuNPs	41	16.7	36.7	27.8	5.6

**Table 3:** The results between the true and predicted value of TBZ in soil.

Model	Sample		Predicted Value (mg/L)			*RSD* (%)	Recovery (%)	
	True Value (mg/L)	Number	Min	Max	Mean		Min	Max
y = 672.26x + 5748.4 (784 cm^−1^)	2	3	1.91	2.33	2.12	8.02	95.90	116.65
	6	3	6.00	6.18	6.07	1.36	100.46	103.16
y = 1155.4x + 8740.2 (1008 cm^−1^)	2	3	1.91	2.22	2.07	5.96	95.73	110.83
	6	3	5.92	6.28	6.08	2.41	98.66	104.59
y = 784.97x + 6935.2 (1270 cm^−1^)	2	3	1.77	2.14	1.92	8.23	88.25	106.78
	6	3	5.19	5.82	5.40	5.45	86.64	96.97
y = 535.17x + 6798 (1328 cm^−1^)	2	3	1.87	2.27	2.11	8.34	93.25	113.33
	6	3	5.01	5.67	5.26	5.58	83.46	94.57
y = 385.33x + 6277.3 (1406 cm^−1^)	2	3	1.73	1.89	1.83	3.84	86.62	94.65
	6	3	4.66	4.76	4.76	3.84	77.66	79.26
y = 607.13x + 6248.3 (1564 cm^−1^)	2	3	1.44	1.71	1.56	7.39	71.87	85.57
	6	3	4.99	6.19	5.39	10.45	83.13	103.19

**Table 4:** The results of pre-processing methods for calibration and prediction model.

Methods	Pre-Processing Method	Calibration		Prediction		
		*R_C_* ^2^	RMSEC (mg/L)	*R_P_* ^2^	*RMSE_P_* (mg/L)	*RPD*
PLS	Original	0.9600	0.607	0.9549	0.694	4.79
	S-G	0.9580	0.622	0.9528	0.691	4.65
	MSC	0.9592	0.647	0.9683	0.678	4.62
	SNV	0.9493	0.711	0.9769	0.556	5.97
biPLS	Original	0.9518	0.698	0.9746	0.523	6.13
	S-G	0.9532	0.684	0.9657	0.605	5.39
	MSC	0.9633	0.600	0.9580	0.683	5.01
	SNV	0.9415	0.741	0.9754	0.561	6.29

**Table 5:** The results between the real values and predicted values of TBZ in red soil extracts.

Sample	Ture Value (mg/L)	Predicted Value (mg/L)	*RSD* (%)	Recovery (%)
1	1	1.12	12	112
2	2	2.119	5.9	105.95
3	2.5	2.253	9.8	90.12
4	5	4.46	10.8	89.2
5	7.5	7.57	0.93	100.93
